# Effect of different rotary instrument designs (protaper ultimate and protaper gold) on postoperative pain and bacterial reduction: a randomized clinical trial

**DOI:** 10.1186/s12903-026-07973-9

**Published:** 2026-03-25

**Authors:** Khaled Hassan Abed, Ahmed Abdel Rahman Hashem, Reem Ahmed Lutfy, Somaia Abdellatif Eissa, Dina Ahmed Morsy

**Affiliations:** 1 Department of Endodontics, Faculty of Dentistry, Cairo University, Cairo, Egypt; 2 Cleveland Dental Institute, Cairo, OH USA; 3 Department of Endodontics, Faculty of Dentistry, Ain Shams University, Cairo, Egypt; 4 Department of Endodontics, Faculty of Dentistry, Cairo University, Cairo, Egypt; 5 Cairo University Faculty of Medicine, Cairo, Egypt; 6 Department of Endodontics, Faculty of Dentistry, Cairo University, Cairo, Egypt

**Keywords:** Analgesics, Bacterial reduction, Instrument design, Postoperative pain, ProTaper gold, ProTaper ultimate

## Abstract

**Background:**

ProTaper Ultimate (PTU) is one of the recently introduced nickel–titanium rotary endodontic systems and possesses distinctive instrument design features characterized by a parallelogram cross-sectional geometry. This design is produced through an alternating offset machining process, resulting in a file whose center of mass is not aligned with its center of rotation. Such configuration is claimed to create increased space for debris removal during instrumentation while simultaneously reducing stress concentration on the instrument during cutting.

Despite the manufacturer’s claims regarding the novel geometry and expected advantages of ProTaper Ultimate files, independent clinical scientific evaluation of these features remains limited. Understanding whether the parallelogram cross-section and offset center of mass truly translate into improved clinical performance and enhanced debris elimination is essential for evidence-based endodontic practice. Therefore, this study aimed to objectively assess these claimed advantages, providing clinicians with reliable data regarding the bacterial reduction and postoperative pain of PTU. The findings will contribute to informed decision-making when selecting rotary systems and may clarify the clinical relevance of this new design concept.

**Aim:**

This prospective randomized double-blinded trial clinically compared postoperative pain and bacterial reduction after using two rotary systems; ProTaper Ultimate (PTU) versus ProTaper Gold (PTG) in necrotic maxillary premolars.

**Methodology:**

Fifty-six patients with necrotic maxillary first premolars with normal periapical condition were randomly assigned to PTU (n=28) or PTG (n=28). Root canal preparation was performed using the allocated file system. The primary clinical outcome was postoperative pain and was recorded preoperatively and at 6, 12, 24, and 48 hours using a modified visual analogue scale (mVAS). The secondary outcome was intracanal bacterial load and bacterial reduction percentage, quantified as aerobic and anaerobic colony-forming units per milliliter (CFU/ml). Samples were collected pre-instrumentation (S1), post-instrumentation (S2), and after final irrigation with 2.5% sodium hypochlorite (NaOCl) (S3). Incidence and number of analgesic intakes was also documented. Data were analyzed using Student’s t-test, chi-square, Mann-Whitney U test, Friedman’s test, frequencies (n), percentages (%), Fisher exact test, repeated measures ANOVA, and Spearman’s correlation coefficient (ρ).

**Results:**

Postoperative pain was significantly higher with PTU than PTG immediately (p=0.004) and at 12 hours (p=0.035), 24 hours (p=0.002), and 48 hours (p=0.005), with no significant difference at 6 hours (p=0.055). Analgesic intake was significantly greater in the PTU group, which was 3.45-fold more prone to require medication. PTU showed significantly lower bacterial reduction than PTG after instrumentation (S1–S2: aerobic p=0.0014; anaerobic p=0.0326) and after final irrigation (S1–S3: aerobic p=0.0183; anaerobic p=0.0276). Spearman’s correlation analysis revealed no significant association between bacterial reduction and pain scores in either group (PTU: ρ = 0.06, p = 0.760; PTG: ρ = 0.03, p = 0.896).

**Conclusion:**

PTG, with its convex triangle cross-section and lower rotational speed and torque, resulted in lower postoperative pain intensity, less need for analgesics and statistically higher bacterial reduction compared to the parallelogram cross-section of PTU.

**Trial registration:**

The study protocol was listed on www.clinicaltrials.gov (ClinicalTrials.gov identifier: NCT05305742) https://clinicaltrials.gov/study/NCT05305742?cond=Necrosis&viewType=Table&term=GOLD&rank=5.

ClinicalTrials.gov identifier NCT05305742 registration date (30/03/2022).

## Introduction

The primary objectives of root canal treatment revolve around eradicating microbes and avoiding apical periodontitis [1–4]. Removal of biofilm from the root canal walls is critical because persistent adherent biofilm is the primary cause of apical periodontitis and endodontic failure, and its effective disruption and removal significantly improves the likelihood of periapical healing and long-term treatment success [5].

Post-operative pain, high degrees of inflammations and flare up frequency have been linked with bacteria and foreign substances being forced into the periapical tissues i.e. Apical debris extrusion [6], that is, governed by factors such as instrumentation techniques, instrument design, kinematics, and irrigation methods [7].

Therefore, different mechanical systems exhibit inconsistent debris extrusion [8] and prepare the canal differently [9] thus reduce intracanal microbial load to varying degrees. Hand instrumentation techniques achieve roughly 40–60% reduction, while machine driven instrumentation techniques can achieve approximately 70–90% reduction according to kinematics; continuous rotary motion typically reduces intracanal bacteria by about 70–85%, while reciprocating motion can achieve approximately 80–90% reduction, neither method ensures complete disinfection without irrigation [10].

The impacts of instrumentation techniques and kinematics were extensively studied [11], but instrument design, particularly cross-section design, remains a topic under investigation [12]. Accordingly, it would appear worthwhile to look more into the instrument design impact, such as cutting blade design, tip type, cross-section, cutting efficiency, taper, flexibility, configuration, alloy, and number of files used [7] specially in files with the same kinematics and instrumentation technique under the same irrigation protocol.

One of the most recent generations of rotary Nickel Titanium (NiTi) file systems, namely ProTaper Ultimate (PTU) files (DENTSPLY, Tulsa Dental, TN) [13]. According to the manufacturer, PTU’s unique parallelogram cross-section geometry, combined with various acute angles along different sections of the instrument, defines the design of PTU. Additionally, according to the manufacturer, the alternating offset machining process allows the files to have a geometry where the center of mass is not aligned with the center of rotation, in which this design increases space for debris removal and reduces stress levels during cutting [14, 15].

Similar in design to the ProTaper Ultimate is the ProTaper Gold (PTG) (DENTSPLY, Tulsa Dental, TN), a heat-treated (NiTi) rotary system. The PTG exhibits two-stage transformation behavior along with a high Austenite finishing temperature (Af) (heat-treated), which results in the advanced metallurgy of PTG for superior flexibility and high cyclic fatigue resistance [16, 17]. Both PTU and PTG systems have the same rotary kinematics and follow the Deep Shape concept of increased apical taper of rotary instruments. Both file systems are nearly the same in file sequence, number, size, variable taper, and tip type. The main difference lies in cross-section geometry and cutting angles [15], in which the PTG files have a convex triangular cross-section instead of a parallelogram.

Up to this date, very few clinical studies [18, 19] have been conducted to assess the impact of the instrument design of the recently launched ProTaper Ultimate rotary system. The previous clinical investigations have reported no significant differences either in bacterial reduction between ProTaper Ultimate and comparable systems or in postoperative pain between ProTaper Ultimate and ProTaper Gold; however, variations in case selection, pulpal and periapical diagnoses, baseline pain levels, and follow-up protocols limit direct comparability, thereby justifying further evaluation under standardized conditions such as those applied in the present trial.

Thus, the purpose of this research was to clinically examine the effect of ProTaper Ultimate compared to ProTaper Gold on the reduction of bacterial load and postoperative pain in subjects with necrotic maxillary premolars after single-visit root canal therapy. The null hypothesis was that there is no difference in bacterial reduction and postoperative pain between the two file systems.

## Methods

### Protocol registration and study design

The Consolidated Standards of Reporting Trials (CONSORT) 2010 guidelines were used in writing this randomized clinical trial. This prospective, intention to treat (ITT), single-center, double-blinded, parallel randomized clinical trial (1:1 allocation ratio) was approved by the Faculty of Dentistry, Cairo University institutional review boards (Code: ENDO: 3-3-5). The protocol was registered on www.clinicaltrials.gov with the NCT number (NCT05305742), registration date (30/03/2022).

### Ethics approval and consent to participate

This randomized clinical trial was approved by the Faculty of Dentistry, Cairo University ethics committees (reference number 23/3/22). The details of the study and treatment have been clearly communicated to participants who were requested to sign a consent form. Throughout the treatment of patients, the Declaration of Helsinki was employed as revised in 2013.

### Patient selection

Patients were recruited from the outpatient clinic of the Faculty of Dentistry and referred for treatment at the Endodontic Clinic, where patients received treatment between 10/04/2022 to 03/05/2025. The details of the study and treatment have been clearly communicated to participants who were requested to sign a consent form. Throughout the treatment of patients, the Declaration of Helsinki was employed as revised in 2013.

### Criteria of eligibility

#### Criteria of inclusion

- Healthy patients with status (ASA I) [20].
- Age range 22 to 45 years old with no gender preference.
- Maxillary permanent first premolar teeth (#5 or #12) were clinically diagnosed as necrotic with normal periapical condition, where:
  - No response to thermal and electrical pulp testers.
  - No or mild pre-operative pain scoring (0–3) according to the mVAS scale [21].
  - No pain on percussion.
  - Radiographic evidence of two roots, each with a Type I mature canal with no/slight widening of the periodontal membrane space.

#### Criteria of exclusion

- Pregnant or lactating females.
- History of intolerance to non-steroidal anti-inflammatory drugs.
- Receiving preoperative medication within 7 days before treatment, such as antibiotics, analgesics, or anti-inflammatory medications.
- Reporting bruxism or clenching.
- One or more adjacent teeth require endodontic treatment.
- Teeth associated with:
  - Vital pulp tissues (Bleeding seen during access cavity preparation).
  - Acute periapical abscess, swelling, or sinus tract.
  - Pocket of more than 5 mm depth or mobility (Grade II or III)
  - History of previous root canal treatment.
- Non-restorable tooth.
- Radiographic proof of immature canal, internal or external root resorption, periapical radiolucency, calcification, vertical root fracture, root caries, or perforation.

### Sample size calculation

According to the study by Aggarwal and Dewan [22], the sample size for assessing postoperative pain at 48 h was calculated using the G*Power program. A total of 50 participants (25 in each group) was deemed adequate to reject the null hypothesis, achieving 80% power at a 5% significance level. To account for potential dropouts, the sample size was adjusted to 56 participants (28 per group), anticipating a dropout rate of 10%. The sample size was determined using version 3.1.9.7 of the power and sample size calculation software developed at Heinrich Heine Universität, Düsseldorf, Germany.

### Clinical examination

Demographic data, along with medical, dental, and chief complaint histories, were taken from all patients through forms that they filled out. Intra- and extra-oral examinations were carried out for each patient. History of no (0) to mild pain (1–3) to (rated on the mVAS scale) was recorded. Each examined tooth expressed a negative response to the cold sensitivity test using Ethyl Chloride Spray (Walter Ritter GmbH + Co. Pharmaceutica – Hamburg, Germany) as well as to the electric pulp test (DY310 Dental Pulp Tester, Denjoy Dental Co, Hunan, China). An intra-oral peri-apical sensor plate and software (Digora; Soredex, Helsinki, Finland) were utilized to conduct radiographic examination. To ensure consistency, all radiographs were standardized using the paralleling technique with a sensor holder and taken using the same X-ray machine (Belray II 097, Belmont, Japan) under fixed exposure parameters: 70 kVp, 8 mA, and 0.15 s. The angulation, sensor position, and focal distance were kept constant across all cases to minimize distortion and allow accurate comparison.

### Randomization, allocation, and blinding

#### Randomization

The random allocation sequence was generated using a computerized randomization tool (www.random.org) to randomly assign subjects to either the control group or the intervention group in a 1:1 allocation ratio by an independent co-investigator not involved in treatment or outcome assessment. The co-investigator knew the table of codes and accordingly packed folded numbered papers from 1 to 56 in opaque sealed envelopes.

#### Allocation concealment

The operator contacted the co-investigator to determine the patient group. Each envelope was opened only after access cavity preparation and confirmation of pulpal necrosis, ensuring that group allocation was concealed and could not be predicted in advance.

#### Blinded outcome assessors

The study employed double blinding at the patient and outcome-assessor levels, while acknowledging that operator blinding was not feasible due to the inherent differences in the rotary instruments and motor settings.

- Microbiological outcome assessment: All bacterial samples were coded immediately after collection and transferred to the microbiology laboratory without group identifiers. Microbiology laboratory personnel were blinded to group allocation throughout culture processing and CFU counting.
- Postoperative pain assessment: Pain scores were self-reported by patients using the mVAS charts at predefined intervals. Patients were unaware of the file system used. Follow-up communication for pain recording was performed using coded patient identifiers, maintaining assessor blinding.
- Statistical analysis: Data were anonymized and coded prior to analysis, and the statistician was blinded to group allocation during data processing and analysis.

### Root canal treatment procedure

Treatment was done in a single visit by the principal investigator. Patients rated their preoperative pain using the mVAS scale. Infiltration technique was used to anaesthetize each tooth with 1.8 ml of local anesthesia; 4% Articaine HCl with 1/100,000 adrenaline (Septodont, Maur-des-Fossés, France) in a 27-gauge short needle. A rubber dam was used for tooth isolation, and all coronal restorations and/or caries were removed. Cleaning of the operative field; tooth, rubber dam sheet, and clamp were performed using 3% hydrogen peroxide (Merck KGaA, Darmstadt, Germany) until bubbling stopped. A sterile cotton swab with a sodium hypochlorite (NaOCl) 2.5% solution (Calix Sodium Hypochlorite, Dharma Research, Miami, Florida) was utilized to disinfect all surfaces. A sterile round carbide bur size #3 (Hager & Meisinger GM, Neuss, Germany) and an Endo-Z bur (DENTSPLY, Tulsa Dental, TN) were used to perform the access cavity. The absence of bleeding in the pulp chamber confirmed the diagnosis of a necrotic pulp. After completing the access, the operative field and pulp chamber were disinfected with NaOCl, followed by 5% sodium thiosulfate.

Patency of each root canal was performed using stainless steel hand K-files #10 (MANI, INC., Industrial Park, Utsunomiya, Tochigi, Japan). Evaluation of pre-instrumentation bacterial count (S1) in each necrotic root canal was carried out. A side-vented needle gauge #30 (CERKAMED, Poland) and a plastic disposable syringe were used to inject 1 ml of sterile saline into each root canal. Three sterile paper-points #15 / 0.02 (Meta Biomed Co., Ltd, Korea) were consecutively placed inside each canal (6 paper points for each tooth) till 1 mm short of apex based on estimated working length and kept for one minute with pumping motion to generate a suspension of bacteria in the main pulpal area [23].Paper points were then immediately placed inside sterile coded tubes containing 1 ml thioglycolate broth transport medium [24] (Thioglycolate broth U.S.P alternative, Oxoid microbiology product, LTD, England) and sent to the microbiology lab within 1 h. An electronic apex locator (Root ZX, J. Morita, Japan) and hand K-files #15 (MANI, INC., Industrial Park, Utsunomiya, Tochigi, Japan) were used to measure the working length, which was confirmed using radiographic imaging.

According to the randomization, mechanical shaping of each tooth was performed by operator according to the file system assigned for its group. Following the manufacturer’s instructions, a gear reduction torque-controlled endodontic motor (X-Smart, Dentsply Maillefer, Switzerland) in a continuous rotary brushing movement was used. PTU rotary files were operated at 400 rpm and 4 Ncm. The following sequence was followed: PTU Slider #16 .02v, PTU Shaper #20 .04v, PTU F1 #20 .07v, ending with PTU F2 #25 .08v to the full working length in both buccal and palatal canals. PTG rotary files were used at speed 300 rpm, and torque(T) was set according to the file size; S1 #18 .02v (T 5.2 Ncm), S2 #20 .04v (T 1.5Ncm), F1 #20 .07v (T 1.5 Ncm), ending with F2 #25 .08v (T 3.1 Ncm). All files and instruments were used as single-use files for each tooth.

During instrumentation of each canal, irrigation with 2 ml saline was carried out before each file and after the last file using a total of 10 ml saline. Post-instrumentation bacterial root canal (S2) was then collected following the same S1 protocol using paper points size #30/ 0.04. The irrigation needle was placed up to approximately 1 mm short of the working length and this depth was standardized for all cases.

Finally, final irrigation of each canal was performed using 25 ml of 2.5% NaOCl with 1 min of Passive Ultrasonic Activation (PUA). Passive Ultrasonic activation (PUA) was performed using Ultramint Pro ultrasonic device (Eighteeth, Iangsu Province, China) with a E1 Tip at power settings set at 1 Endo Mode. The tip was positioned passively at approximately 3 mm short of working length without contacting canal walls.

This was followed by 10 ml of saline flush, followed by 5 ml of 17% EDTA (Meta Biomed Co., Ltd, Korea) with 1 min of PUA and ending with 10 ml saline flush [25]. Post-final irrigation bacterial root canal sample (S3) was collected using the same technique as (S2).

Obturation of the root canals was performed after radiographic confirmation of the master cone fit. Modified single cone obturation technique was performed using the PTU Conform Fit #F2 Gutta Percha (DENTSPLY, Tulsa Dental, TN) in the PTU group and the PTG Conform Fit #F2 Gutta Percha (DENTSPLY, Tulsa Dental, TN) in the PTG group. Gutta-percha points #20/ 0.02 (Meta Biomed Co., Ltd, Korea) were used as auxiliaries with ADSEAL resin-based root canal sealer (Meta Biomed Co., Ltd, Korea). Postoperative radiographs were taken to appraise the quality of obturation. The access cavity was sealed using resin-modified high-viscosity glass ionomer (SDI Limited, Bayswater, Victoria, Australia) to ensure an adequate coronal seal. Any subjects who experienced iatrogenic errors, such as over-instrumentation or over-extended obturation, was excluded from the study, to ensure accurate assessment of postoperative pain.

All participants received postoperative instructions to document postoperative pain degree on the mVAS scale at 6, 12, 24, and 48 h postoperatively. Upon onset of severe or persistent pain, one tablet of Ibuprofen 400 mg as a post-operative analgesic was prescribed to be taken at 6-hour intervals and to be recorded. Participants recorded the incidence and number of analgesic intake if needed during the 48 h following treatment. The patients were contacted at each time interval to check on them and as a reminder for documentation. Patients were asked to return after 48 h to return charts with pain and analgesic recorded.

### Bacterial count procedure

Each sample (6 paper points for the 2 canals of each tooth in 1 ml of thioglycolate broth transport medium) was dispersed and vortexed in the mixer (Vortex Mixer, OHAUS Europe GmbH, Nänikon, Switzerland) for 30 s. Then 100 µl (µl) of the vortexed sample was placed in a new sterile tube, which contained 1 ml of thioglycolate broth using a micropipette, accordingly, making a 1/10 dilution sample.

To prepare aerobic and anaerobic bacterial cultures, 50 µl of the diluted sample was dispersed over brain heart infusion (BHI) agar plate (Brain Heart Infusion Agar, Oxoid microbiology product, LTD, England). For aerobic conditions, the plates were cultivated under aseptic conditions before incubation at 37 °C for 24 h. For anaerobic conditions, the plates were cultivated under aseptic conditions and placed in an anaerobic sealed jar with GasPak (Anaerogen gas pack AN0035, Oxoid microbiology product, LTD, England) and anaerobic indicator (Anaerobic indicator BR0055B, Oxoid microbiology product, LTD, Basingstoke, Hampshire, England) followed by incubation for 48 h at 37°C [26].

The number of colony-forming units per millilitre (CFU/ml) of each sample was counted manually, with magnification assistance using a laboratory microscope when required to improve visualization of small or closely adjacent colonies [27] to measure the resulting bacterial growth on the agar medium. Colony enumeration was performed by a single trained examiner using a standardized protocol. Utilizing one evaluator ensured methodological consistency and avoided inter-observer variability.

Accordingly, using the previously established dilution factors, the number of colonies counted on agar plate was subsequently translated into actual counts using the following equation:

$$\begin{aligned}\mathrm{CFU/ml} &= \text{Number of Colonies} \times \text{Dilution Factor} \times\\& \text{Total Amount of Sample} / \text{Amount Put on Agar Plate}\end{aligned}$$

$$\mathrm{CFU/ml} = \text{Count on petri dish} \times 1/10 \times 1000\; \mu \text {l} / 50 \; \mu \text {l}$$

$$\mathrm{CFU/ml} = \text{Count on petri dish} \times 10 \times 20$$

Then absolute aerobic and anaerobic bacterial counts were analyzed and expressed as log₁₀ CFU/ml at S1, S2, and S3. These data were used for statistical analysis. The percent bacterial reduction was calculated using the below formula and compared between the two groups.

$$\% \; \text{bacteria reduction} = \left[\left( \mathrm{S1}-\text{S2 or S3} \right)\right] \times 100$$

The percent bacterial reduction was computed separately for aerobic and anaerobic cultures for S1–S2, S2–S3, and S1–S3 comparisons.”

**Fig. 1:**
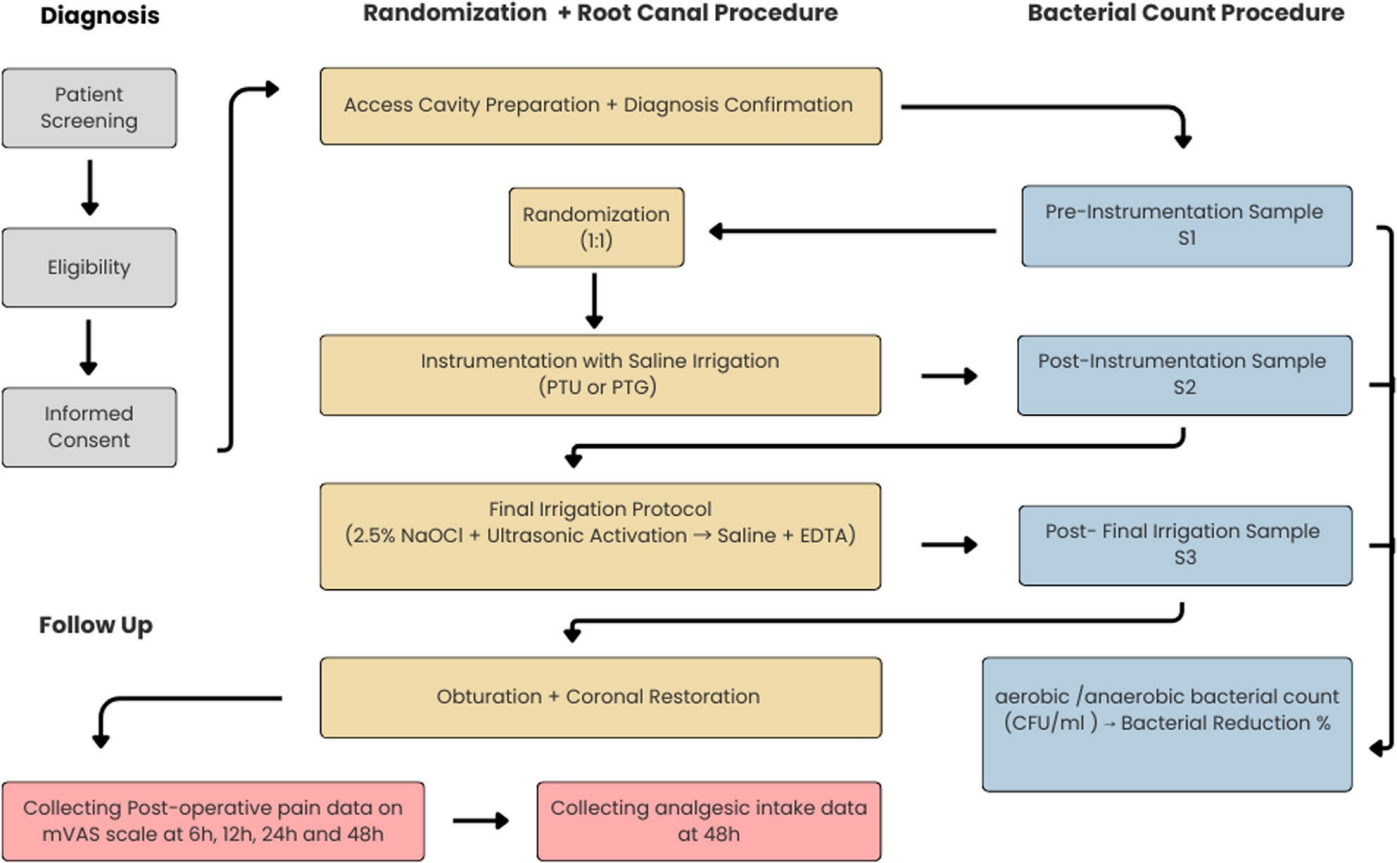
Trial Design Scheme (Participant Timeline)

To enhance reproducibility and improve visualization of the randomized control trial, a concise trial design scheme is illustrated in (Fig. 1) to demonstrate the sequence of microbiological sampling (S1, S2, S3) and postoperative pain assessment intervals.

### Statistical analysis

IBM SPSS Statistics for Windows, Version 23.0. Armonk, NY; IBM Corp was utilized to collect and analyze the data. To demonstrate normality, the Shapiro-Wilk and Kolmogorov-Smirnov tests were utilized. Regular (parametric) distribution was observed in gender, age, and percentage reduction of bacterial counts, while non-parametric distribution was observed for pain scores and Log_10_ CFU/ml of bacterial counts.

To compare age and gender, the Chi-square test and the student’s t-test were used. The Mann-Whitney U test and descriptive statistics were utilized to perform a comparison of pain (mVAS scores), number of analgesic tablets consumed and log_10_ CFU/ml bacterial counts. Descriptive statistics and results of Friedman’s test and descriptive statistics were used to compare the pain intensity changes within each group. The incidence of analgesic intake in the two groups was compared using frequencies (n), percentages (%), and Fisher exact test. To compare the percentage decrease in bacterial counts (%) in the two groups, the outcomes of the repeated measures ANOVA test and descriptive statistics were used. The correlation between percentage reduction in bacterial counts and pain scores was calculated using Spearman’s correlation coefficient (ρ).

## Results

**Fig. 2:**
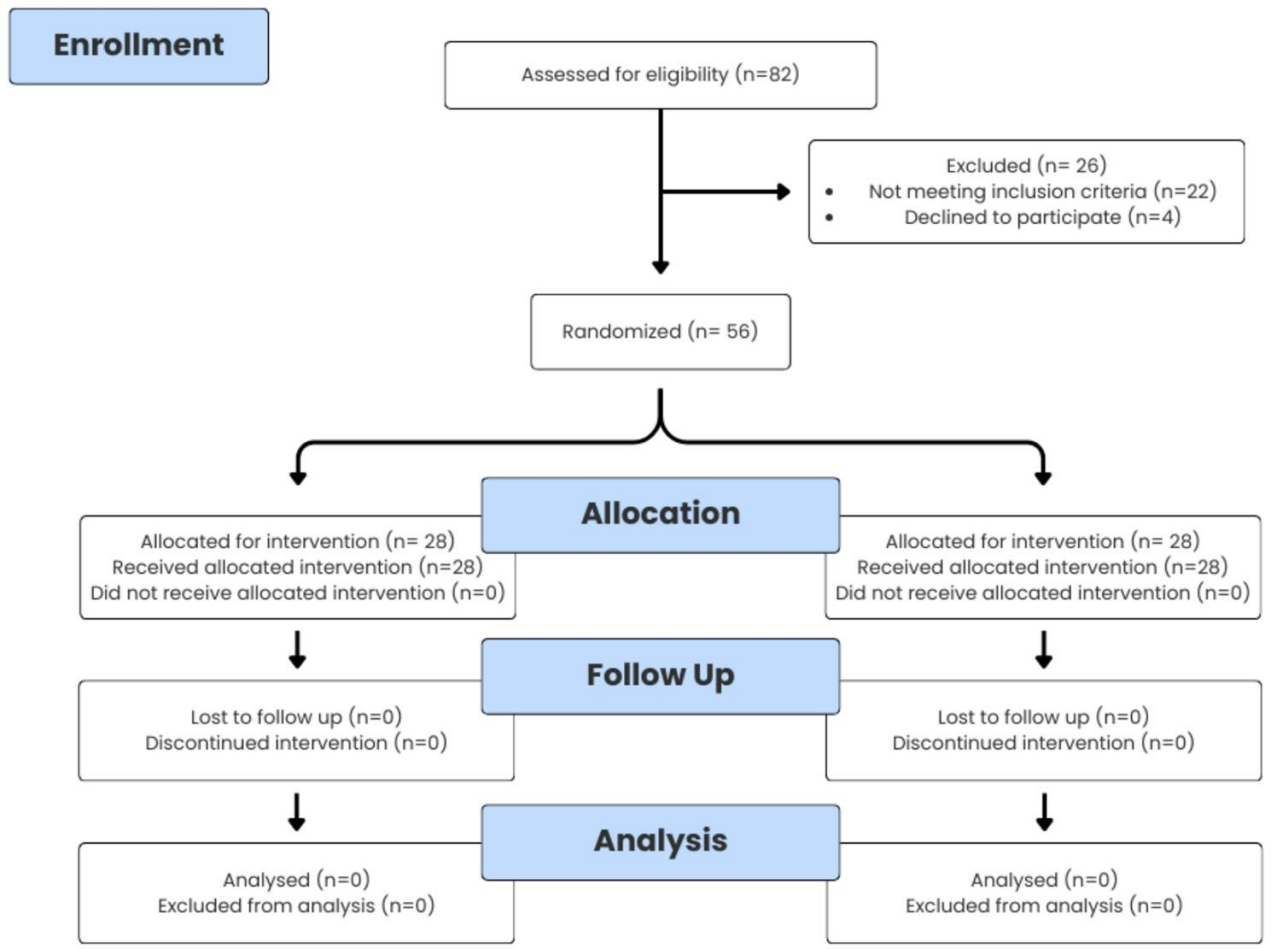
CONSORT 2010 Flow diagram of the trial design

Eighty-two (82) patients were assessed for eligibility, but only 56 patients fulfilled the inclusion criteria and were signed up for the research. They were randomly assigned to one of the two groups. The analysis included every patient **(**Fig. 2**).**

Demographic data are presented in (Table 1), showing no statistical difference in gender and age between the 2 groups.

**Table 1:** Descriptive statistics and results of the Chi-square test and Student’s t-test for comparisons between baseline characteristics in the two groups

Base line characteristics	PTU (*n* = 28)	PTG (*n* = 28)	*P*-value
Gender [*n*, (%)]			
Male	10 (35.7%)	13 (46.4%)	0.415
Female	18 (64.3%)	15 (53.6%)	
Age [Mean, SD]	35.3 (5.6)	33.2 (5.8)	0.164

* Significant at *P* ≤ 0.05

The results of pain intensity are shown in **(**Table 2**)**. Pre-operatively, there was no significant difference between the two groups. PTU has shown statistically significantly higher postoperative pain intensity scores compared to PTG immediately (*p* = 0.004) and at 12 (*p* = 0.035), 24 (*p* = 0.002), and 48 h (*p* = 0.005) postoperatively. At 6 h, there was no discernible statistical difference (*p* = 0.055).

**Table 2:** Descriptive statistics and results of the Mann-Whitney U test for comparison between pain (mVAS scores) in the two groups

Time	PTU (*n* = 28)		PTG (*n* = 28)		*P*-value	Effect size (d)
	Median (Range)	Mean (SD)	Median (Range)	Mean (SD)		
Pre-operative	0 (0, 3)	0.57 (0.96)	0 (0, 3)	0.5 (1)	0.514	0.136
Immediate post-operative	4.5 (0, 8)	3.59 (2.35)	2 (0, 7)	1.75 (1.9)	0.004*	0.821
6 h	2.5 (0, 9)	3.29 (2.99)	1 (0, 7)	1.93 (2.43)	0.055	0.518
12 h	2 (0, 10)	3.5 (3.67)	0 (0, 10)	1.93 (3.22)	0.035*	0.54
24 h	1.5 (0, 9)	2.79 (3.19)	0 (0, 6)	0.75 (1.76)	0.002*	0.805
48 h	0 (0, 6)	0.86 (1.9)	0 (0, 0)	0 (0)	0.005*	0.439

* Significant at *P* ≤ 0.05

The results of Friedman’s test and descriptive statistics for the changes within each group are shown in (Table 3).

**Table 3:** Descriptive statistics and results of Friedman’s test for the changes within each group

Time	PTU (*n* = 28)		PTG (*n* = 28)	
	Median (Range)	Mean (SD)	Median (Range)	Mean (SD)
Pre-operative	0 (0, 3) ^D^	0.57 (0.96)	0 (0, 3) ^C^	0.5 (1)
Immediate post-operative	4.5 (0, 8) ^A^	3.59 (2.35)	2 (0, 7) ^A^	1.75 (1.9)
6 h	2.5 (0, 9) ^B^	3.29 (2.99)	1 (0, 7) ^A^	1.93 (2.43)
12 h	2 (0, 10) ^B^	3.5 (3.67)	0 (0, 10) ^B^	1.93 (3.22)
24 h	1.5 (0, 9) ^C^	2.79 (3.19)	0 (0, 6) ^C^	0.75 (1.76)
48 h	0 (0, 6) ^D^	0.86 (1.9)	0 (0, 0) ^C^	0 (0)
*P*-value	< 0.001*		< 0.001*	
*Effect size (w)*	0.192		0.235	

* Significant at *P* ≤ 0.05,Different superscripts in the same column indicate statistically significant change by time

In PTU group, there was a statistically significant change in pain scores by time (*P*-value < 0.001, Effect size = 0.192). Pair-wise comparisons between time periods revealed that there was a statistically significant increase in pain scores immediately post-operative followed by a statistically significant decrease in pain scores after six hours. From six to 12 h, there was no statistically significant change in pain scores. This was followed by a statistically significant decrease in pain scores from 12 to 24 as well as 24 to 48 h. Pain scores after 48 h showed non-statistically significant difference from pre-operative scores.

**Fig. 3:**
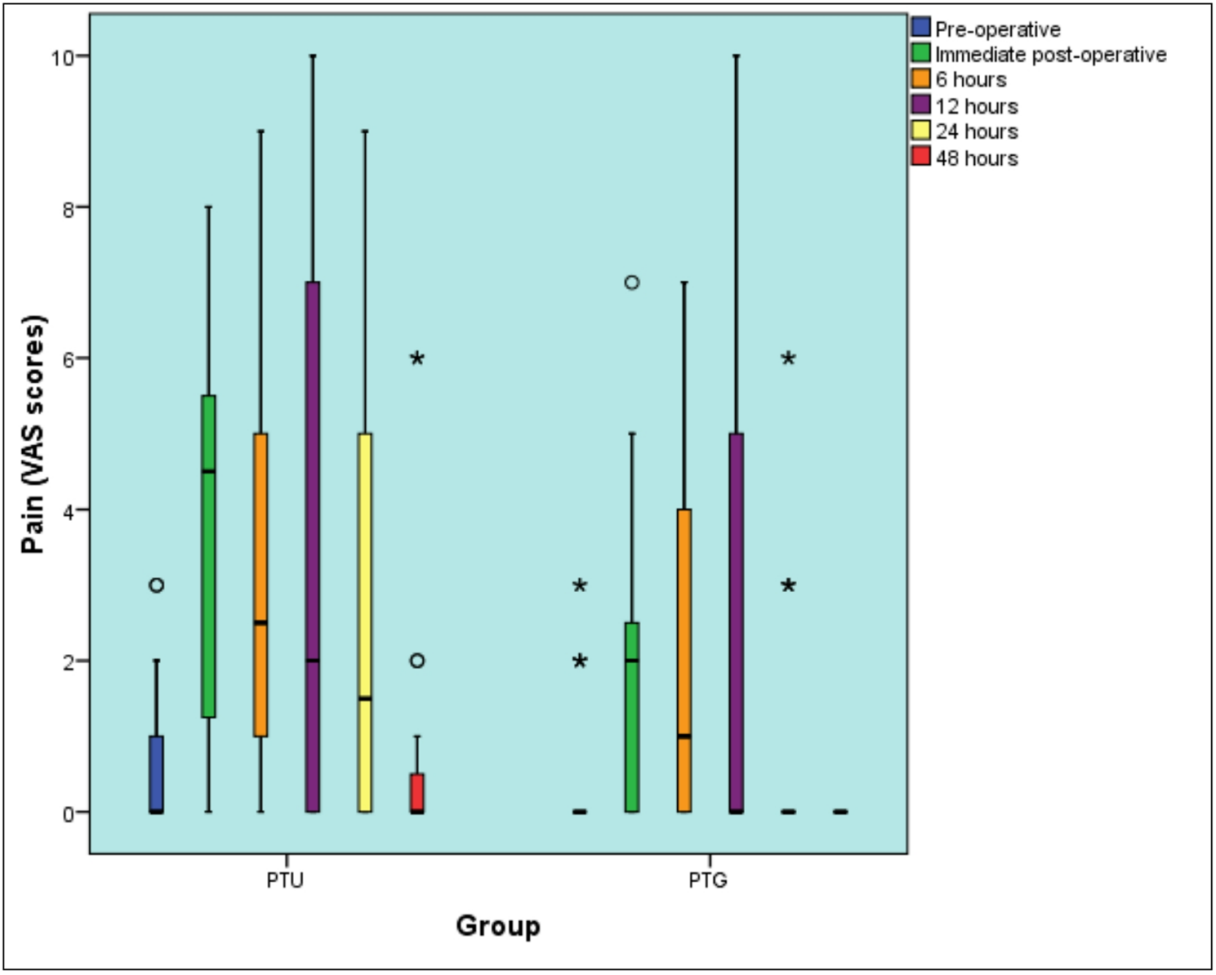
Box plot representing median and range values for pain scores in the two groups (Stars and circles represent outliers)

In PTG group, there was a statistically significant change in pain scores by time (*P*-value < 0.001, Effect size = 0.235). Pair-wise comparisons between time periods revealed that there was a statistically significant increase in pain scores immediately post-operative followed by non-statistically significant change in pain scores after six hours. From six to 12 as well as 12 to 24 h, there was a statistically significant decrease in pain scores followed by non-statistically significant change in pain scores from 24 to 48 h. Pain scores after 48 h showed non-statistically significant difference from pre-operative scores. Figure 3

**Fig. 4:**
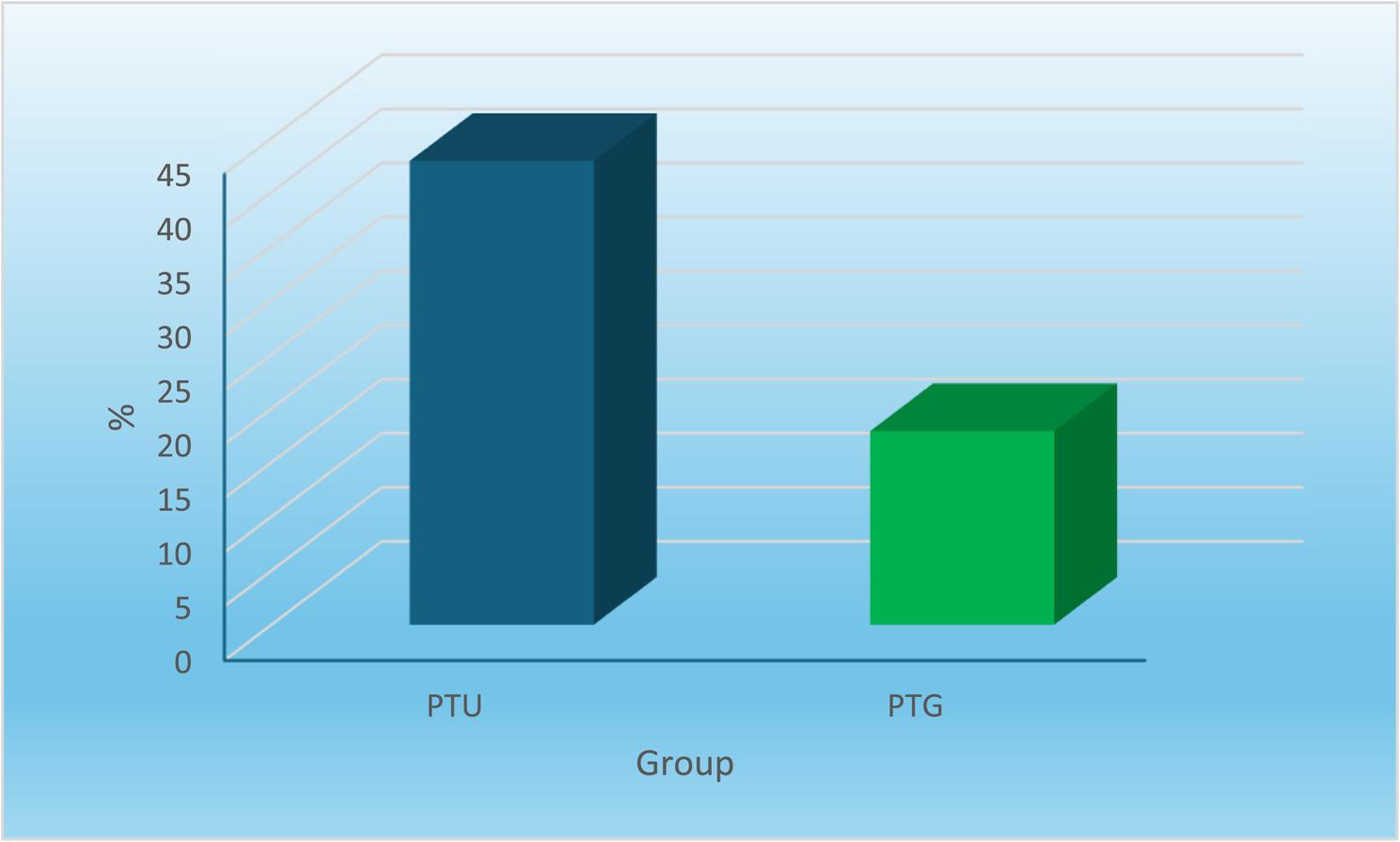
Bar chart representing intake of analgesics in the two groups

Incidence of analgesic intake is expressed in (Table 4) and (Fig. 4), PTU group showed statistically significantly higher prevalence of incidence of analgesics intake than PTG group (*P*-value = 0.046, Odds Ratio = 3.45). PTU group is 3.45 times more prone to use analgesics than PTG group.

**Table 4:** Frequencies (n), percentages (%) and results of Fisher exact test for comparison between analgesics intake in the two groups

	PTU (*n* = 28)		PTG (*n* = 28)		*P*-value	Odds Ratio (95% CI)
	*n*	%	*n*	%		
Intake of analgesics	12	42.9	5	17.9	0.046*	3.45 (1.02–11.72)

* Significant at *P* ≤ 0.05

Number of analgesic tablets taken in the two groups is presented in (Table 5). On day one, PTU group showed statistically significantly higher number of analgesics than PTG group (*P*-value = 0.023, Effect size = 0.72). On day two, there was no statistically significant difference between the two groups (*P*-value = 0.081, Effect size = 0.48). As regards the total number of analgesics, PTU group showed statistically significantly higher number of analgesics than PTG group (*P*-value = 0.023, Effect size = 0.74).

**Table 5:** Descriptive statistics and results of Mann-Whitney U test for comparison between number of analgesic tables consumed in the two groups

Time	PTU (n = 28)		PTG (n = 28)		P-value	Effect size (d)
	Median	Range	Median	Range		
Day 1	0	0 - 3	0	0 - 1	0.023*	0.72
Day 2	0	0 - 1	0	0 - 0	0.081	0.48
Total	0	0 - 3	0	0 - 1	0.023*	0.74

* Significant at *P* ≤ 0.05

Bacterial count is presented in (Table 6) presents in the form of log10 CFU/ml values and is compared between the two groups at the three sampling stages. For aerobic bacteria (A), baseline counts (S1) were significantly higher in the PTG group compared with PTU (*p* = 0.00002), with a large effect size (d = − 1.46). After instrumentation with saline irrigation (S2), bacterial counts decreased markedly in both groups and the intergroup difference was no longer statistically significant (*p* = 0.093). Following the final irrigation protocol (S3), very low bacterial levels were observed in both groups, with no significant difference between PTU and PTG (*p* = 0.155). For anaerobic bacteria (AN), no statistically significant difference was detected between the groups at baseline (S1) (*p* = 0.059). Similarly, at S2 the bacterial counts were comparable (*p* = 0.807). After final irrigation (S3), both systems achieved near-complete reduction, and the difference between groups remained statistically non-significant (*p* = 0.054).

**Table 6:** Descriptive statistics and results of Mann-Whitney U test for comparison between Log_10_ CFU/ml of aerobic and anaerobic bacterial counts in the two groups

Group	PTU (n = 28)		PTG (n = 28)		P-value	Effect size (d)
	Median Log_10_(Range)	Mean Log_10_ (SD)	Median Log_10_(Range)	Mean Log_10_ (SD)		
Aerobic						
S1	1.72 (1.11 – 2.29)	1.72 (0.32)	2.37 (1.59 – 2.53)	2.18 (0.32)	0.00002	-1.46
S2	0.695 (0.44 – 1.01)	0.72 (0.18)	0.775 (0.43 – 1.10)	0.81 (0.20)	0.093	-0.48
S3	0.03 (0 – 0.07)	0.03 (0.02)	0.02 (0 – 0.06)	0.02 (0.02)	0.155	0.46
Anaerobic						
S1	1.91 (1.30 – 2.48)	1.91 (0.32)	2.26 (1.48 – 2.40)	2.07 (0.31)	0.059	-0.52
S2	0.925 (0.68 – 1.36)	0.90 (0.17)	0.875 (0.53 – 1.20)	0.91 (0.2)	0.807	-0.07
S3	0.03 (0.01 – 0.19)	0.07 (0.06)	0.03 (0.01 – 0.11)	0.04 (0.03)	0.054	0.53

* Significant at *P* ≤ 0.05

The reduction percent (%) of aerobic bacteria (A) and anaerobic bacteria (AN) are presented in (Table 7). The percent bacterial reduction between the two groups showed statistical difference at S1-S2 (*p* = 0.0014) (A) and (*p* = 0.0326) (AN), and at S1-S3 (*p* = 0.0183) (A) and (*p* = 0.0276) (AN), while at S2-S3 (*p* = 0.0677) (A) and (*p* = 0.0539) (AN) there was no statistical difference.

**Table 7:** Descriptive statistics and results of repeated measures ANOVA test for comparison of aerobic and anaerobic percent bacterial reduction (%) between the two groups

Group	PTU (n = 28)		PTG (n = 28)		P-value	Effect size (Partial Eta squared)
	Mean %	SD	Mean %	SD		
Aerobic						
S1 - S2	58.15	5.59	63.19	5.07	0.0014*	0.175
S2 – S3	95.76	3.89	97.50	2.22	0.0677	0.061
S1 – S3	98.29	1.41	99.10	0.75	0.0183*	0.099
Anaerobic						
S1 - S2	52.30	8.05	56.21	4.94	0.0326*	0.082
S2 – S3	91.68	7.97	94.95	3.68	0.0539	0.067
S1 – S3	95.98	3.95	97.80	1.61	0.0276*	0.087

* Significant at *P* ≤ 0.05

**Fig. 5:**
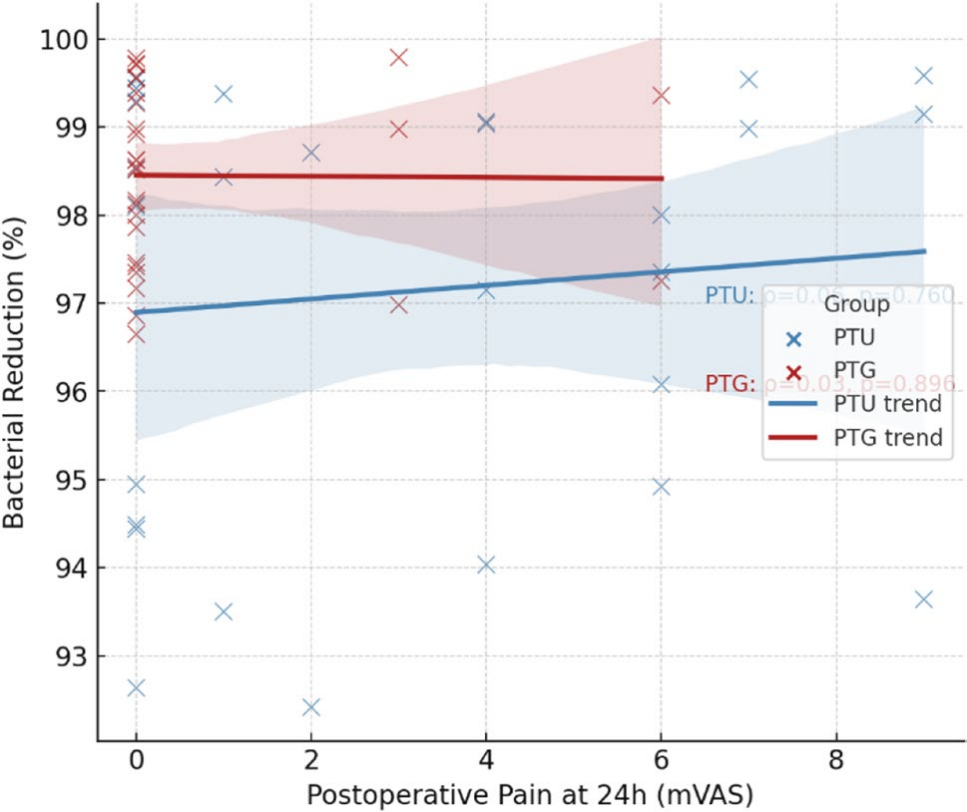
Results of Spearman’s correlation coefficient (ρ) for the correlation between combined bacterial reduction (mean of S1-S3 Aerobic and Anaerobic) versus pain intensity for PTU and PTG

A correlation analysis was performed between pain intensity at 24 h versus combined bacterial reduction % (mean of S1-S3 Aerobic and Anaerobic) as demonstrated in (Fig. 5). The trend lines are almost flat in all cases, suggesting a weak correlation and independency between pain intensity and bacterial reduction in either group. Spearman’s correlation analysis revealed no significant association between bacterial reduction and pain scores in either group (PTU: ρ = 0.06, *p* = 0.760; PTG: ρ = 0.03, *p* = 0.896). Overall bacterial reduction is strong in both groups regardless of the pain intensity at the time.

## Discussion

After a thorough systematic online search, most studies carried out to evaluate the effect of instrument design of the recently launched ProTaper Ultimate rotary systems were either in vitro studies [28–30] or case series [31] and reports [32, 33]. Hence, the main goal of this research was to conduct a clinical randomized controlled trial to examine the effect of the instrument design of ProTaper Ultimate on bacterial elimination and postoperative pain in single-visit treatment of cases with necrotic maxillary premolars. At the outset of our analysis, statistical analysis revealed sufficient evidence to reject the null hypothesis, indicating that the PTG had less postoperative pain and more significant bacterial reduction than the PTU.

One of the principal reasons for selecting ProTaper Gold (PTG) as the comparator for ProTaper Ultimate (PTU) was the substantial similarity between the two systems. Both instruments operate using continuous rotary kinematics, follow a comparable file sequence and number, and are developed under the same shaping philosophy described as the *Deep Shape Concept*. In addition, they exhibit closely related taper characteristics and tip-size progression. These similarities make the comparison clinically meaningful, as it allows evaluation of performance differences between two systems designed within the same conceptual framework rather than between fundamentally dissimilar instrumentation strategies.

However, PTU and PTG differ in rotational speed, torque protocols, and file size of initial file (PTU Slider #16 .02v versus PTG S1 #18 .02v). Therefore, the present study cannot truly isolate cross-sectional design as the single determinant of the observed differences. Our findings reflect the overall performance of the instrument design of each system which part of it includes differences in cross section geometry.

The selected postoperative assessment times (6, 12, 24, and 48 h) were chosen to capture the typical temporal pattern of post-endodontic pain. Previous clinical studies have shown that pain commonly develops within the first few hours after treatment, often peaks during the first 24 h, and then gradually subsides. Therefore, these intervals allow evaluation of the onset, peak intensity, and early resolution of symptoms, while remaining practical for patient follow-up. These intervals capture the clinically most relevant timeframe for patient experience [34–36].

The current research focused on necrotic teeth due to the high prevalence of microorganisms, which are known to cause periapical tissue breakdown and inflammatory effects [37]. The presence of bacteria and endotoxins in these teeth is a significant factor in post-endodontic pain [38, 39]. Patients with pulpal necrosis experience more postoperative discomfort and flare-ups compared to those with vital pulps [40, 41].

Culture-based quantification of bacteria using aerobic and anaerobic incubation was selected because it remains a well-established and clinically interpretable method for assessing viable microorganisms within the root canal system. Unlike molecular techniques, CFU analysis specifically reflects bacteria capable of replication, which are directly relevant to the persistence of infection and potential postoperative symptoms. Furthermore, the use of both aerobic and anaerobic conditions allows recovery of the dominant cultivable microbiota typically encountered in necrotic canals and has been widely employed in clinical endodontic trials evaluating the antibacterial effectiveness of instrumentation and irrigation procedures.

We acknowledge, however, that culture methods may underestimate the total microbial diversity because certain species may be non-cultivable or exist in a viable-but-non-culturable state. For this reason, CFU values should be interpreted as representing the cultivable fraction of the infection rather than the entire microbiome. Future studies are encouraged to integrate molecular diagnostic techniques such as qPCR and 16 S rRNA analysis that would provide complementary information.

Intra-canal sampling using sterile paper points was selected because it is a standard, minimally invasive, reproducible approach and compatible with chairside workflow while still providing quantitative information about intracanal bacterial load. Paper points can be placed to the established working length, allowing absorption of intracanal contents and recovery of cultivable microorganisms from the main canal lumen without introducing additional variables (e.g., dentin removal) that may confound intergroup comparisons. The technique is also practical and ethically appropriate for clinical trials, enabling repeated sampling at multiple stages (S1/S2/S3) under standardized conditions. We acknowledge that this method primarily reflects the planktonic/lumen bacterial fraction and may under-represent bacteria located within dentinal tubules or biofilm structures. Consequently, CFU values should be interpreted as an estimate of recoverable bacteria rather than the total microbial burden [42].

Analyzing the course of the reported pain in both groups, PTU group resulted in significantly higher postoperative pain scores than PTG at all studied time intervals except at 6 h probably due to the ongoing local anesthesia effect at that time. The significant difference in postoperative pain might be attributed to the cross-section and file design of the PTU files, along with its higher rotational speed and torque that might have led to a higher amount of apical debris extrusion thus increased postoperative pain [43].

While PTU and PTG files have almost the same file sizes and same progressive variable taper, they differ in the cross-section and file design. PTU files have a rhomboid cross-section from D1-D3 then evolve as an ever-changing parallelogram from D4-D16 [14]. PTG files, on the other hand, possess convex triangular cross-section along the whole file length [16]. Due to such difference in cross-section design, PTU files possess more cutting blades and more acute helical angles [44], example: PTU Shaper (20/.04v) has 18 blades and 21.9° angle, the corresponding PTG S2 (20/.04v) only has 11 blades and 22.1° angle [44]. Similarly, PTU F1 (20/.07v) is presented with 16 blades and 18.4° angle compared to the corresponding PTG F1 (20/.07v) that has only 12 blades and 25.3° angle [44]. PTU F2 (25/.08v) possesses 16 blades and 19.1° angle compared to the corresponding file PTG F2 (25/.08v) that only has 10 blades and 21.9° angle [44].

Therefore, PTU files possessing more cutting blades may be suspected to result in more apical debris extrusion than PTG files and, hence, more significant postoperative pain. This could explain why the PTU group was 3.45-fold more prone to patients taking analgesics than the PTG group. The current results agree with Al Omari et al. [43], suggesting that the off-centered geometry of PTU files, which was supposedly designed to provide more space for debris clearing, was associated with higher debris extrusion. Similarly, Elmsallati et al. [45], found that the off-centered geometry design resulted in files with more acute helical angles and longer pitch distance, which was associated with more debris extrusion. Sharawy and El Shater [46], also found that PTU files removed significantly more resin than PTG files from resin canals in blocks at 1 mm from the apex.

In contrast, Hajwel et al. [19] in a recent clinical study published in October 2025 stated that there was no significant difference in postoperative pain between patients instrumented with ProTaper Ultimate and ProTaper Gold. Such different results are justified as Hajwel’s clinical study was different in methodology in terms of criteria of patient selection and duration of recording of postoperative pain. The study was conducted on mandibular molar with symptomatic irreversible pulpitis and symptomatic apical periodontitis with a mean preoperative pain according to VAS scale: 7.6 +/- 2.6 for PTG group and 7.9 +/-1.55 for PTU group. Also, the postoperative pain was recorded on a wider duration interval (Preoperative, 24-, 72-h and 7-day intervals).

Bacterial samples were taken pre-instrumentation (S1), post-instrumentation with saline irrigation (S2), and after NaOCl irrigation (S3). Separating S2 from S3 was crucial since NaOCl could mask the effect of instrument design on bacterial reduction. Siqueira et al. [37] and Carvalho et al. [47] reported that saline irrigation revealed significant differences between instrumentation techniques, whereas NaOCl irrigation showed no such differences.

The PTU group showed less bacterial reduction percent than the PTG group at S1-S2 and S1-S3. This could be attributed to the increased number of cutting blades [44] and file design of the PTU, which might have led to the formation of thick and dense debris [43] that would have plugged dentinal tubules and blocked the flushing of the irrigant inside; saline in S2 or NaOCL in S3, attenuating the bacterial reduction effect. This comes in line with results reported by Üreyen et al. [48], where scanning electron microscopic examination revealed that the file that caused the thickest and densest debris caused dentinal tubule plugging, significantly decreasing the bacterial reduction effect of the instrument.

Bacterial reduction from S2 to S3 was borderline and not statistically significant, indicating similar NaOCl effectiveness in both groups. Therefore, the main difference at S1–S3 stems from instrument design effects at S1–S2. While irrigation is considered more vital than instrumentation, the two are interdependent. Effective irrigation relies on proper instrumentation to create a pathway for irrigant delivery and the removal of debris [49].

Although statistical differences were detected in bacterial reduction, both systems produced very low bacterial counts after completion of the irrigation protocol. Therefore, the clinical magnitude of difference should be interpreted with caution, and both approaches may be considered effective in achieving substantial microbial reduction.

A recent clinical study published in October 2025 by kumari et al. [18], compared the bacterial reduction in primarily infected root canals using ProTaper Ultimate (PTU) and ProTaper Next (PTN) systems. The results showed no statistical significance difference between both rotary file systems, which could be justified as both file systems have similar off-centered quadrilateral cross-section instrument design: PTU has off-centered parallelogram cross section while PTN has off-centered rectangular cross section. Therefore, both files systems possess similar number of cutting blades and in turn result in similar amount of debris and bacterial reduction.

The results also showed a weak correlation between pain intensity and bacterial reduction in either condition (Aerobic or Anaerobic) in both groups. Bacterial reduction remained high regardless of pain level. Such correlation is in agreement with Emara et al., who also stated “A weak correlation existed between postoperative pain severity and bacterial counts” [50].

One of the limitations of this study is the use of different paper point sizes at various sampling stages, which was necessary due to canal enlargement following instrumentation. Although this may influence bacterial recovery between time points, identical sampling protocols were applied in both groups, thereby limiting its impact on between-group comparisons. Moreover, a dedicated chemical neutralizing agent for sodium hypochlorite (NaOCl) was not applied immediately before S3 sampling, nor incorporated into the transport medium. Although canals were flushed with sterile saline prior to paper-point collection, residual irrigant activity cannot be completely excluded and may have influenced absolute CFU values, particularly after final irrigation. Therefore, results at S3 and calculations of S1–S3 reduction should be interpreted with caution and reflect the cultivable fraction under these standardized conditions. However, as the identical irrigation and sampling protocol was used in both groups, any potential carryover effect would have affected them equally, preserving the validity of intergroup comparisons. Although, strict aseptic procedures were implemented, including rubber dam isolation and operative field disinfection, a formal negative control (e.g., a sterile paper point exposed to the operative field after disinfection) was not used. The absence of such a control may limit verification of complete environmental sterility. Finally, samples from both canals of each tooth were pooled into a single specimen. While this approach reflects the overall intracanal microbial burden relevant to clinical outcome, it reduces canal-level resolution and may mask potential intra-tooth variability.

It shall be noted that because PTU and PTG differ in rotational speed, torque protocols, and file size of initial file—the present study cannot truly isolate cross-sectional design as the single determinant of the observed differences. Our findings reflect the overall performance of the instrument design of each system which part of it includes differences in cross section geometry. Also, to be noted, that the initial bacterial load in clinical infections cannot be experimentally standardized because it reflects natural biological variability. For this reason, treatment efficacy was primarily expressed as the magnitude of bacterial reduction relative to each tooth’s baseline.

Although the absence of both long-term clinical and radiographic follow-up limits the comparison between the file systems in both groups, this study proved that instrument design may impact postoperative pain and bacterial load reduction. The PTG file system exhibited superior results than the PTU file system in the previously mentioned aspects, therefore endodontists should carefully ponder whether PTU files are the right choice for their treatment.

## Conclusion

Within the limitations of this study, it can be concluded that ProTaper Gold files not only led to lower postoperative pain and analgesic consumption but also demonstrated statistically significant higher bacterial reduction when compared to ProTaper Ultimate files.

## Data Availability

All data supporting the findings of this study are available within the article. Raw data that support the findings of this study are available from the corresponding author, upon reasonable request [dina.amorsy@dentistry.cu.edu.eg].

## Abbreviations

A: Aerobic Bacteria
Af: Austenite Finishing temperature
AN: Anaerobic Bacteria
ANOVA: Analysis of Variance
ASA: American Society of Anesthesiologists’ physical status classification system
BHI: Brain Heart Infusion
CFU/ml: Colony-Forming Units per Millilitre
CONSORT: Consolidated Standards of Reporting Trials
EDTA: Ethylene-diamine-tetra-acetic acid
m-VAS: Modified Visual Analogue Scale
NaOCL: Sodium Hypochlorite
NiTi: Nickel Titanium
PTG: ProTaper Gold
PTN: ProTaper Next
PTU: ProTaper Ultimate
PUA: Passive Ultrasonic Activation
